# SNP discovery in proso millet ( *Panicum miliaceum* L.) using low‐pass genome sequencing

**DOI:** 10.1002/pld3.447

**Published:** 2022-09-13

**Authors:** Rituraj Khound, Guangchao Sun, Ravi V. Mural, James C. Schnable, Dipak K. Santra

**Affiliations:** ^1^ Department of Agronomy and Horticulture University of Nebraska‐Lincoln Lincoln NE USA; ^2^ UNL Panhandle Research and Extension Center Scottsbluff NE USA; ^3^ Center for Plant Science Innovation University of Nebraska‐Lincoln Lincoln NE USA

**Keywords:** ancient grain, climate‐resilient, panicoid, C4 photosynthesis, phylogeny, population structure

## Abstract

Domesticated ~10,000 years ago in northern China, Proso millet ( *Panicum miliaceum* L.) is a climate‐resilient and human health‐promoting cereal crop. The genome size of this self‐pollinated allotetraploid is 923 Mb. Proso millet seeds are an important part of the human diet in many countries. In the USA, its use is restricted to the birdseed and pet food market. Proso millet is witnessing gradual demand in the global human health and wellness food market owing to its health‐promoting properties such as low glycemic index and gluten‐free. The breeding efforts for developing improved proso millet cultivars are hindered by the dearth of genomic resources available to researchers. The publication of the reference genome and availability of cost‐effective NGS methodologies could lead to the identification of high‐quality genetic variants, which can be incorporated into breeding pipelines. Here, we report the identification of single‐nucleotide polymorphisms (SNPs) by low‐pass (1×) genome sequencing of 85 diverse proso millet accessions from 23 different countries. The 2 × 150 bp Illumina paired‐end reads generated after sequencing were aligned to the proso millet reference genome. The resulting sequence alignment information was used to call SNPs. We obtained 972,863 bi‐allelic SNPs after quality filtering of the raw SNPs. These SNPs were used to assess the population structure and phylogenetic relationships among the accessions. Most of the accessions were found to be highly inbred with heterozygosity ranging between .05 and .20. Principal component analysis (PCA) showed that PC1 (principal component) and PC2 explained 19% of the variability in the population. PCA also clustered all the genotypes into three groups. A neighbor‐joining tree clustered the genotypes into four distinct groups exhibiting diverse representation within the population. The SNPs identified in our study could be used for molecular breeding and genetics research (e.g., genetic and association mapping, and population genetics) in proso millet after proper validation.

## 1 INTRODUCTION

Proso millet (*Panicum miliaceum* L.) is one of the oldest cereal crops known to mankind. It was domesticated approximately 10,000 years ago in the semiarid regions of northern China (Hunt et al., 2014; Lu et al., 2009). Following domestication, the crop spread westward across the Eurasian steppes, being widely cultivated in eastern Europe by 3000 BC (Valamoti, 2016). This minor millet is cultivated on ~820,000 and 700,000–1,000,000 ha of farmland in Russia and China, respectively (Vetriventhan et al., 2020; Wang et al., 2016). German–Russian immigrants brought proso millet seeds with them when they migrated to the United States (Habiyaremye et al., 2017; Santra, 2013; Wietgrefe, 1990) and started early cultivation along the Atlantic coast of North America, which later spread westward into the interior of the continent (Wietgrefe, 1990). Today, proso millet is widely cultivated across the High Plains of the United States, with approximately 200,000 ha of annual production (Santra et al., 2019).

Proso millet is prized for both its short growing season (60–90 days) and high water‐use efficiency (Briggs & Shantz, 1913; Nielsen & Vigil, 2017). The shallow root system of proso millet, combined with its high water‐use efficiency, producing the most grain from the least water of any crop, means that the crop leaves behind more soil moisture for subsequent crops (Nielsen & Vigil, 2018). Proso millet's rapid life cycle and water sparing nature have made it a desirable rotation partner with winter wheat where it can replace a summer fallow, enabling farmers to harvest an additional crop of grain from the same field (Agdag et al., 2001; Anderson et al., 1999; Lyon et al., 2008; Hinze & Smika, 1983; Halvorson et al., 1994).

Proso millet is an essential part of the human diet in many countries in Asia, Europe, and Africa including India, China, South Korea, Japan, and eastern Europe (Carpenter & Hopen, 1985; Das et al., 2019). However, in the USA, the largest single consumer of proso millet is the birdseed and pet food market, with significant additional demand coming from the export and gluten‐free health food markets (Graybosch & Baltensperger, 2009; Habiyaremye et al., 2017; Kalinova & Moudry, 2006). The proso millet protein contains a specific prolamin fraction below permissible level, making it a suitable low‐GI (glycemic index), gluten‐free diet for patients suffering from celiac disease (Kalinova & Moudry, 2006; McSweeney et al., 2017). Proso millet seeds are a good source of carbohydrates, protein, fat, and crude fiber (Motta Romero et al., 2017; Saleh et al., 2013; Vetriventhan & Upadhyaya, 2018). Some phenolic compounds in the seed are known to protect against cancer and heart disease (Kalinová, 2007). All these nutritional and health‐promoting properties make proso millet an excellent food for people suffering from serious diseases like type‐2 diabetes mellitus, cardiovascular disease, and celiac disease (Santra et al., 2019). In recognition of both its climate‐friendly and human health‐promoting properties, Food and Agricultural Organization (FAO) listed proso millet as one of the future smart crops of the 21st century (Li & Siddique, 2018).

The proso millet genome is relatively small (923 megabases) (Zou et al., 2019). However, the species is an allotetraploid with a chromosome number of 2*n* = 4*x* = 36 (Hamoud et al., 1994). The two subgenomes of proso millet are estimated to have diverged from each other ~5.6 million years ago (Zou et al., 2019). Substantial genetic redundancy exists between the subgenomes of proso millet with partial redundancy between the two duplicated copies of the GBSSI gene (Hunt et al., 2013).

Genomic resources in proso millet are very limited compared to the major crops as it is largely an under‐researched and underutilized crop (Upadhyaya et al., 2014). Understanding the population structure of crop germplasm based on genetic diversity analysis is important for genetic improvement and marker‐trait association studies. In proso millet, genetic diversity of the available germplasm has been studied using phenotypic (morphological, agronomical, and seed nutrients) and genotypic data (Hunt et al., 2011; Johnson et al., 2019; Li et al., 2021; Rajput & Santra, 2016; Upadhyaya et al., 2011; Vetriventhan et al., 2019). Prior to the recent genome sequence (Shi et al., 2019; Zou et al., 2019), the genomic resources of proso millet were limited to non‐sequence‐based DNA markers (e.g., RAPD, AFLP, and SSR) (Khound & Santra, 2020; Santra et al., 2019) and sequence‐based single nucleotide polymorphisms (SNPs) (Johnson et al., 2019; Rajput et al., 2016; Wang et al., 2018; Yue et al., 2016). Since the whole genome was published, several populations of proso millet lines have been genotyped for larger numbers of SNPs using either restricted‐site associated DNA (RAD‐seq) to genotype 2,412 segregating SNP markers or specific‐locus amplified fragment (SLAF‐seq) to genotype up to 126,822 SNP markers (Boukail et al., 2021; Li et al., 2021). Boukail and coworkers identified 2,412 SNPs from 494 million reads generated by RAD‐seq from 88 accessions of varieties and landraces. They used those SNPs to study the diversity of the population and perform GWAS. They detected 13 marker‐trait associations (MTAs) for several agronomic and seed traits. Li et al. more recently discovered 126,822 filtered SNPs using SLAF‐seq of 106 accessions (9 weedy from China and 97 cultivated accessions mostly from East Asia and a few from other regions such as Europe, West Asia, and India), to study genetic diversity and population structure. The genetic diversity indices, that is, observed heterozygosity (H_o_), expected heterozygosity (H_E_), and nucleotide diversity (π), of the cultivated proso millet were significantly lower than the weedy types. The wild and feral types of weedy proso millet could also be distinguished using the SNPs (Li et al., 2021).

Both RAD‐seq and SLAF‐seq employ approaches to target a part of the genomes for sequencing. In principle, this reduces the total quantity of sequence data that must be generated from each individual. However, both approaches increase the complexity of the molecular biology steps necessary to generate libraries for sequencing and necessarily restrict the total number of markers that can be discovered. Here, we employ low coverage whole‐genome resequencing to characterize a set of 85 proso millet accessions originating from around the globe. Specifically, we seek to (1) identify segregating markers across within the representative sample of global proso millet germplasm (~700 genotypes), including all the US cultivars, which were not sampled in previous studies, and (2) quantify patterns of population structure and phylogenetic relationships among the proso millet lines using the identified SNPs.

## 2 MATERIALS AND METHODS

### 2.1 Plant material (genotypes and growth conditions)

**FIGURE 1:**
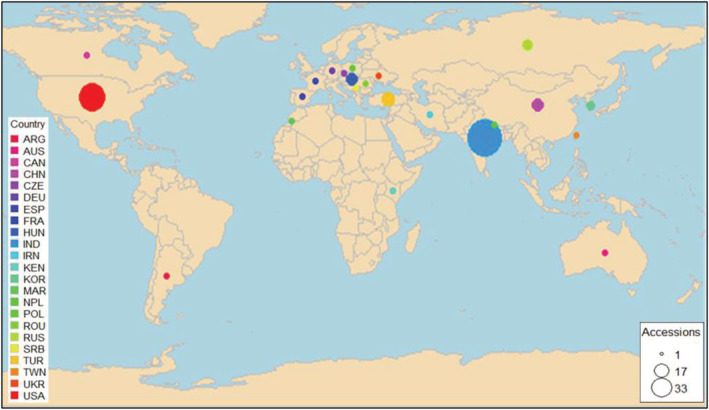
Global distribution of the 85 proso millet accessions used in the study. The accessions belonged to South Asia, 34; East Asia, 7; West Asia, 6; North America, 19; Europe, 15; and others (Argentina, Kenya, Morocco, and Australia), 4. The map was created using the "rworldmap" package in R.

A set of 85 proso millet accessions from 23 different countries (Table S1; Figure 1) along with one accession of *Panicum hallii* (used as an outgroup) were planted in an experimental plot at the UNL Panhandle Research and Extension Center, Scottsbluff, Nebraska, USA (coordinates: 41°53′27″N 103°40′39″W, 1,200 m elevation). The genotypes were planted on a conventionally tilled ground with Tripp very fine sandy loam (fine silty, mixed, and mesic Aridic Haplustolls) type soil (Rajput et al., 2016). The plot was maintained in accordance with the agronomic practices recommended for proso millet (Lyon et al., 2008).

### 2.2 DNA extraction and Illumina sequencing

Leaf samples for DNA extraction were harvested at phenological stage 9 on the BCCH scale after 4 weeks of planting (Ventura et al., 2020). Leaves were cut into 4 cm (~80 mg) pieces and harvested in 2 ml microcentrifuge tubes. The DNA from the leaf tissues was extracted using the MagMAX Plant DNA Isolation kit following the manufacturer's protocol (Applied Biosystems, Massachusetts, USA). The eluted DNA samples were purified using a 96 Deep‐Well KingFisher Flex Magnetic Particle Processor (Thermo Fisher Scientific, Waltham, Massachusetts, USA). The purity of the extracted DNA was quantified using a DS‐11 FX^+^ spectrophotometer/fluorometer (DeNovix). Library construction for sequencing was conducted using the iGenomX RIPTIDE high throughput rapid library prep kit (iGenomX, Carlsbad, California, USA) following the manufacturer's instructions (https://igenomx.com/product/riptide/). The samples were subsequently sequenced to an average coverage depth of 1× utilizing 2 × 150 bp paired‐end reads on the Illumina HiSeq X platform by Psomagen Inc., Rockville, Maryland, USA.

### 2.3 Data preprocessing and sequence alignment

The resulting paired‐end sequence data were quality checked using the FastQC software v .11.2 (Babraham Bioinformatics, https://www.bioinformatics.babraham.ac.uk/projects/fastqc/). Low‐quality sequences and adapters were removed from the sequence data using Trimmomatic software (v .33) with parameter settings “PE Illuminaclip: ~dir/contaminants.fasta: 2:30:10 SLIDINGWINDOW:4:15 HEADCROP:8 MINLEN:101 CROP:100” (Bolger et al., 2014). The surviving trimmed reads were aligned to version 1 of the proso millet reference genome (Zou et al., 2019) using bowtie2 v 2.3 with default parameters (Langmead & Salzberg, 2012). The SAM files were then converted to sorted BAM files using samtools v 1.9 (Li et al., 2009). Picard v 2.22 was used to mark the duplicated reads resulting from PCR amplification.

### 2.4 SNP calling and filtering

The sorted BAM files generated after sequence alignment were used to call SNPs using GATK toolkit v 5.1 (Schmidt, 2009). The resulting raw SNPs were filtered to remove SNPs with maximum missing values of 40% of samples as well as those with a minor allele frequency (MAF) less than .1. In other words, SNPs with at least 60% data (60% or more genotypes) were kept and remaining were not included in the analysis. The missing sites in the filtered SNPs were imputed with Beagle v 5.1 with parameter settings “window = 1, overlap = 0.1, ne = 1,200” (Browning et al., 2018).

### 2.5 Data analysis

Allele frequency data were tabulated from the VCF‐formatted data using −freq option in plink v1.90 (Purcell et al., 2007). The data were plotted using standard Minor Allele Frequency (MAF) plots in python package seaborn. The heterozygosity, and homozygosity count of each allele at a SNP (with MAF > .05) over all genotypes, was calculated using −freqx option in plink v1.90, and the results were plotted using python package seaborn. Heterozygosity per genotype/line over all SNPs > .05 MAf were calculated using −het option from vcftools v0.1.16 (Danecek et al., 2011), and plotting was done in python package seaborn. Principle component analysis (PCA) was performed using TASSEL v5 (Bradbury et al., 2007), and the eigenvalues of 10 PCs were selected to create the scree plot to determine the proportion of variance explained by each PC. Further, PC1 was plotted against PC2 for the PCA plot in python package seaborn. A neighbor‐joining phylogenetic tree was constructed from the distance between each taxon/genotype for SNPs with MAF > .05 using a TASSEL plugin “−tree Neighbor” (TASSEL v5). The phylogenetic tree was displayed using iTOL, an online tool for displaying and annotating phylogenetic tree (Letunic & Bork, 2021). Each genotype was color coded following the same coloring scheme as was used in the PCA plot. To determine the relatedness among 13 of the 85 accessions with pedigree information, a SNP‐based kinship matrix was estimated using the "‐Kinship" plugin in Tassel v5, and was visualized as a heatmap using the "gplots" package in R software. The R statistical package “ggplot2” was used to generate the supplementary bar diagram depicting sequence alignment rates of all the accessions.

## 3 RESULTS

The total number of 150 bp paired‐end reads from sequencing 85 proso millet accessions was ~632 million, amounting to more than 86 billion bp. The number of the 150 bp paired‐end reads and total sequence of each of 85 genotypes are in Table S1. The mean and median of 150 bp paired‐end reads among the 85 accessions were 7 million. The mean and median for the number of sequenced base pairs (bp) were 1,017 and 960 million, respectively. The average genome coverage was 1.1×. The number of 150 bp paired‐end reads following quality control and trimming per accession ranged from 2.4 to 18 million (Figure S1). The maximum number of 150 bp paired‐end reads was 18 million in PS32 (PI 463141), followed by 16 million in PS48 (PI 463263). The long reads of sequence beyond 150 bp and low repeat content of the proso millet genome enabled a large proportion (97.6%) of total quality trimmed sequence reads to be successfully aligned to the proso millet reference genome (Figure S2).

**FIGURE 2:**
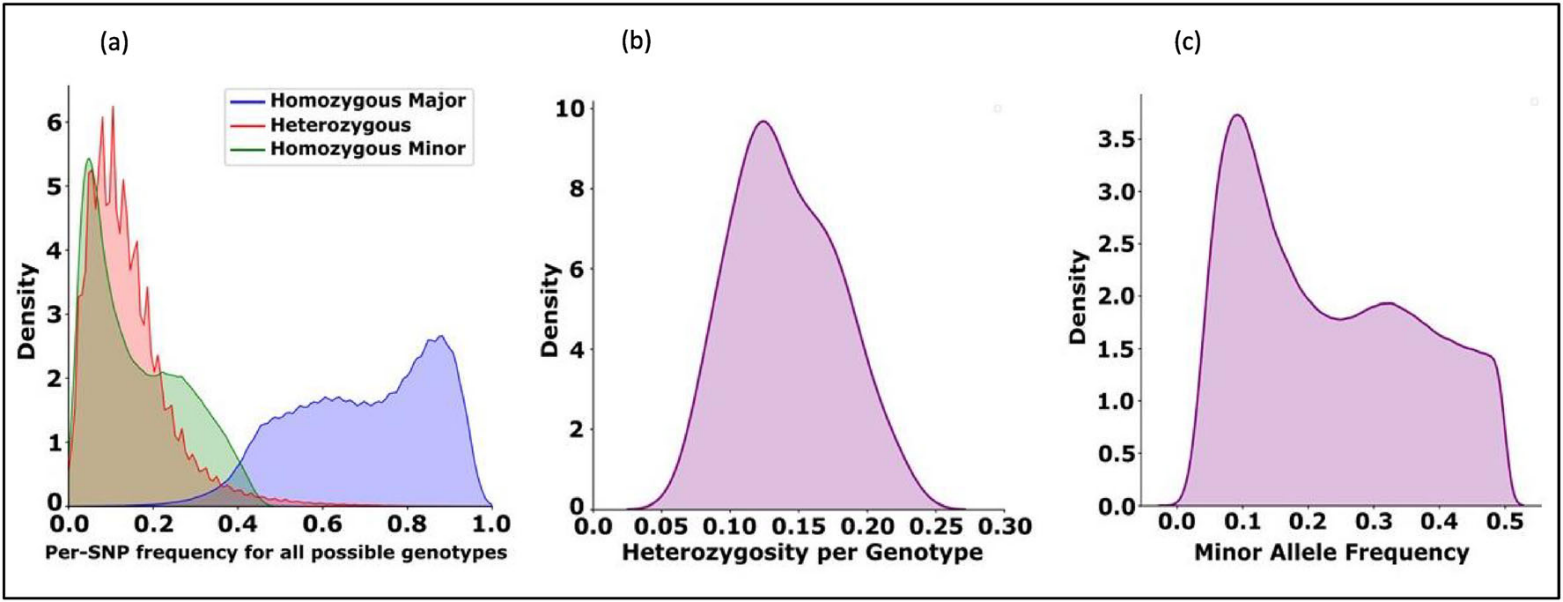
Analysis of the 972,863 filtered SNPs identified in the study. (a) Frequency distribution of homozygous (major and minor) and heterozygous alleles of the individual SNPs for all genotypes. (b) Frequency distribution of heterozygosity of individual genotypes. (c) Distribution of minor allele frequencies (MAFs) of the genotypes

Initial SNP calling identified 9,675,218 SNPs of which 972,863 bi‐allelic SNPs survived quality filtering, representing an average density of approximately one genetic marker per 950 base pairs. SNPs were distributed across all 18 chromosomes based on the Manhattan plot (figure not included) of proso millet with higher SNP densities observed in the gene‐rich chromosome arms than in the more repeat‐rich chromosome arms. The number of SNPs per 10 Mb bins ranged from 0 (all chromosomes) to 1,850 (chromosome 6). The frequency distribution of the homozygous major, homozygous minor, and heterozygous allele for each SNP over all genotypes is shown in Figure 2a. Most of the accessions appeared to be highly inbred. The heterozygosity was between .05 and .20 (Figure 2b). This is expected because of the self‐compatible reproduction (self‐pollination) of proso millet. The proportion of individuals' homozygous minor alleles ranged from 0 to .45 (Figure 2c).

A large proportion of the apparent heterozygosity may be explained by “paralogous” SNPs generated from incorrect alignments between duplicated sequences (Bukowski et al., 2018) including those resulting from the whole genome duplication in the ancestor of proso millet. Paralogous SNPs exhibited high degrees of apparent heterozygosity across majority accessions and few or no examples of the homozygous minor allele. While most SNPs were heterozygous in less than 10% of genotypes, a significant number were heterozygous in between 10–80% of genotypes. This subset of apparent heterozygous SNPs likely results from the alignment of paralogous sequences, which caused the overall frequency of heterozygosity across all individuals in the dataset.

**FIGURE 3:**
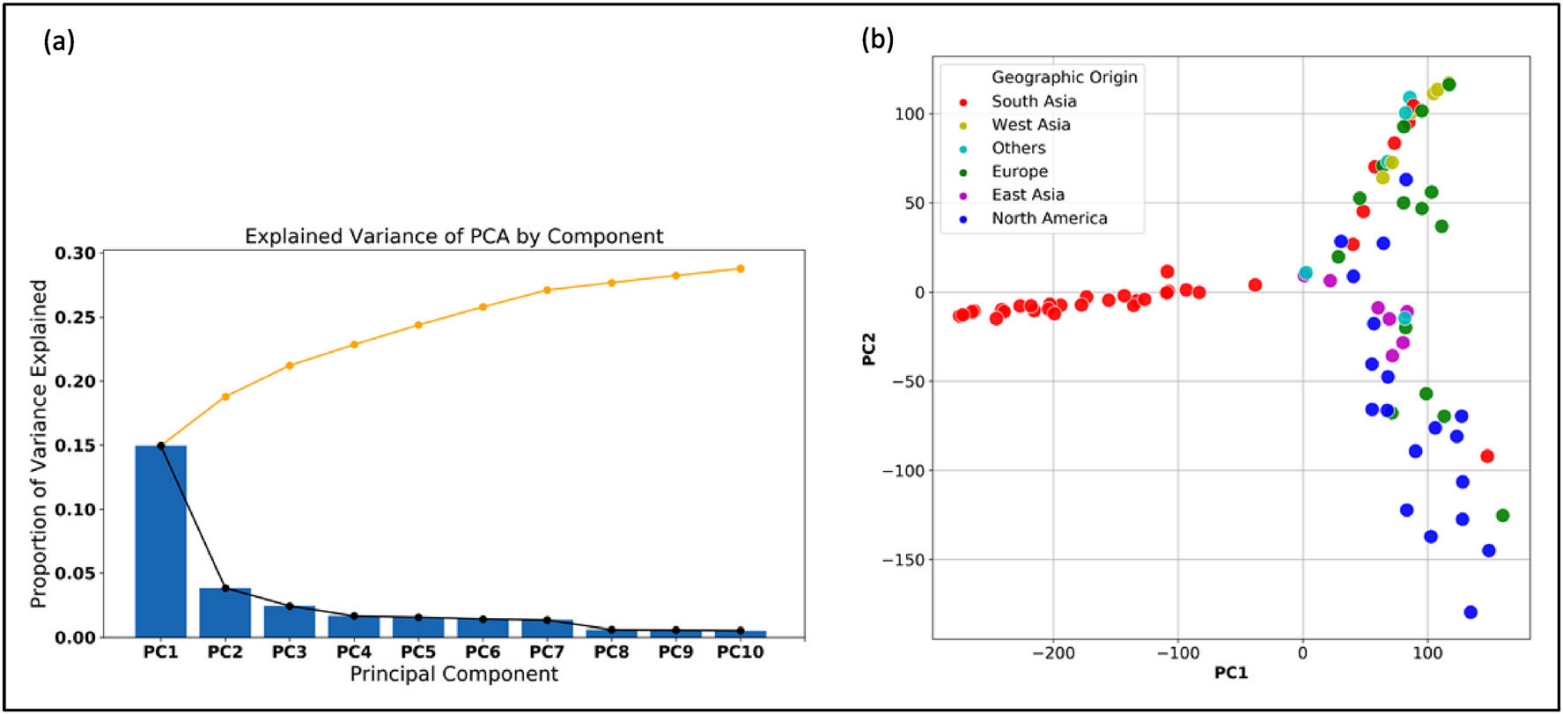
Principal component analysis (PCA) of the filtered SNPs depicting the population structure among the 85 proso millet lines. (a) Total variation explained by 10 principal components (PCs). (b) The relationship among all the accessions exhibited by a score plot of the first (PC1) and second (PC2) principal components. The colors correspond to the geographical origins of the lines. The numbers of accessions in top‐left, bottom‐right, and top‐right of the plot were 27, 23, and 35, respectively.

Principal component analysis (PCA) of the filtered SNPs generated 10 principal components (PCs), and the total variation explained by the 10 PCs is ~33% ranging from 15% (PC1) to ~1% (PC10) (Figure 3a). The first two PCs explained ~19% of the variation observed in SNPs. PC1 explained 15% of the variation while PC2 explained 4%. A score plot of PC1 and PC2 showed the relationship among the 85 accessions (Figure 3b). The majority of the South Asian accessions (27) clustered together on the plot (top left). Another cluster comprised of 23 genotypes predominantly of North American accessions along with accessions from East Asia and Europe (bottom right). A third cluster was formed by 35 accessions from all the geographical regions (top right).

**FIGURE 4:**
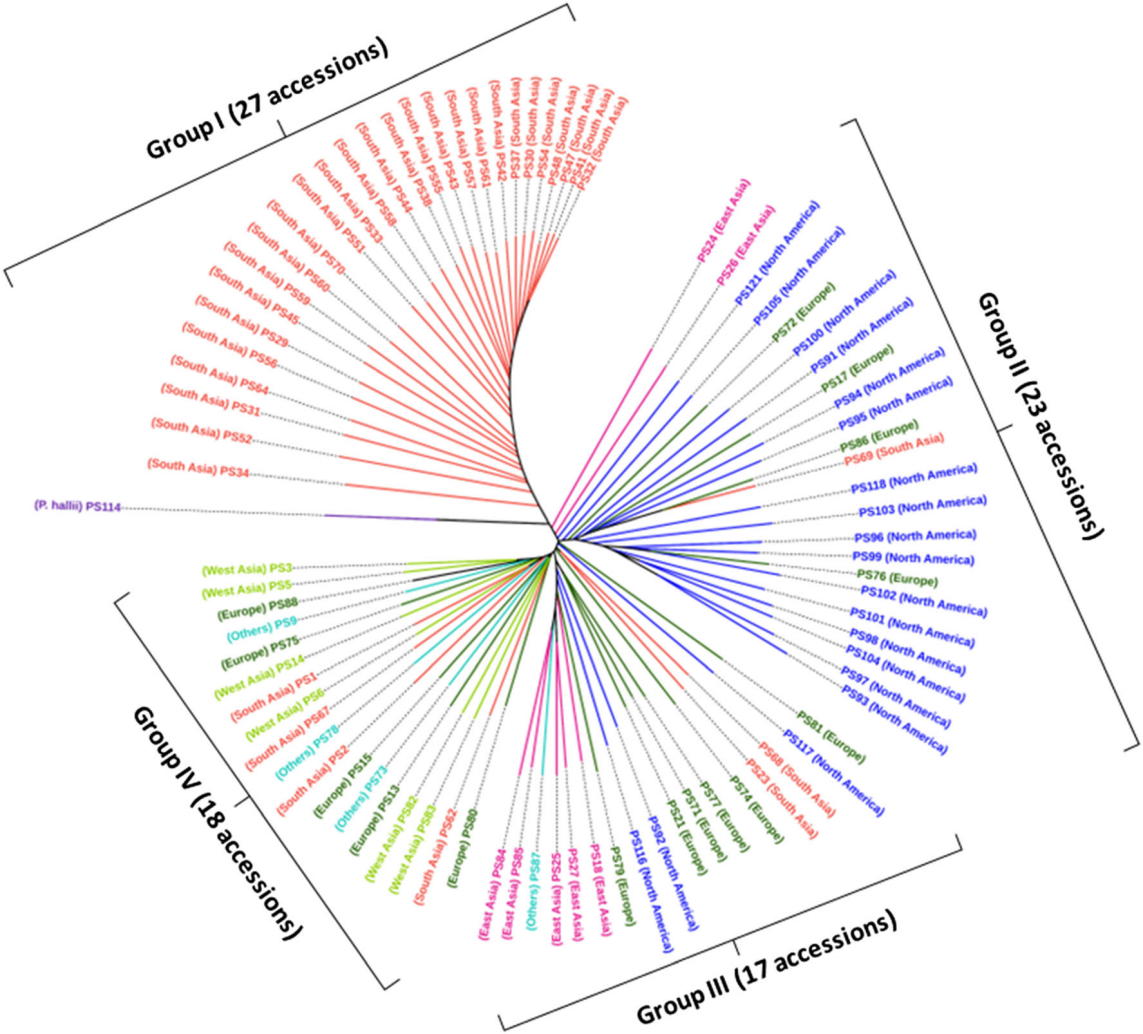
Phylogenetic relationship among the 85 proso millet lines genotyped as part of the current study. A neighbor joining tree constructed from 972,863 SNPs, with a single *P. hallii* accession (PS114) used as an outgroup. Colors indicate geographic origin of each accession and follow the color convention established in Figure 3b.

A phylogenetic tree demonstrated the relatedness of the 85 proso millet accessions (Figure 4). The majority of the South Asian lines (27), all from India, were grouped together (Group I). The second cluster (Group II) consisted of 23 accessions included 16 of the 19 North American, 2 East Asian (PS24 and PS26), 1 Indian (PS69), and 4 European accessions (PS17, Hungary; PS72, Romania; PS76, Germany; and PS86, Russia). The third cluster (Group III) included the remaining five (PS18, PS25, PS27, PS84, and PS85) of the seven East Asian, two South Asian (PS23, Nepal, and PS68, India), six European (PS21, Ukraine; PS71, Russia; PS74, Hungary; PS77, Russia; PS79, Poland; and PS81, Hungary), three North American (PS92, PS116, and PS117), and one Australian (PS87). A fourth group consisted of 18 accessions including six West Asian and 14 from all the geographical regions except East Asia and North America (Group IV). Accessions in this group were six West Asian (Iran, PS14; Turkey, PS3, PS5, PS6, PS82, and PS83), four South Asian (PS1, PS2, PS62, and PS67, all from India), five European (PS13, Serbia; PS15, Spain; PS75, Czech; PS80, Hungary; and PS88, France), and three from others (PS9, Argentina; PS73, Morocco; and PS78, Kenya). It is important to note that all six West Asian genotypes (Turkey, 5; Iran, 1) were grouped in Group IV.

Both the score‐plot (PCA) based cluster analysis (Figure 3b) and neighbor‐joining tree (Figure 4) essentially illustrated the same relationship among the 85 accessions except the group of 35 lines. All the 27 accessions of the top left and 23 lines of the bottom right group in score‐plot cluster analysis (Figure 3b) remained together in the neighbor‐joining tree (Figure 4) represented by Groups I and II in the neighbor‐joining tree, whereas the 35 accessions of the top‐right cluster in Figure 3b were divided into two distinct clusters of 17 genotypes (Group III) and 18 genotypes (Group IV) in the neighbor‐joining tree.

To verify the reliability of the SNPs, we analyzed the neighbor‐joining phylogenetic tree and a heatmap depicting relatedness among the genotypes in detail. We observed that almost all the genotypes of the same gene pool (geographical origin) were grouped together in the phylogenetic tree (Figure 4), and the following are the supporting examples.

- Twenty‐seven of 34 South Asian genotypes were grouped together in Group I. The IDs of these 27 genotypes are: PS29, PS30, PS31, PS32, PS33, PS34, PS37, PS38, PS41, PS42, PS43, PS44, PS45, PS47, PS48, PS51, PS52, PS54, PS55, PS56, PS57, PS58, PS59, PS60, PS61, PS64, and PS70 (Table S1).
- All the six West Asian genotypes (PS3, PS5, PS6, PS14, PS82, and PS83) were placed together in Group IV.
- Five of seven East Asian genotypes (PS18, PS25, PS27, PS84, and PS85) were grouped together in Group III and other two (PS24 and PS26) were placed in Group II.
- Sixteen of 19 North American genotypes (PS91, PS93, PS94, PS95, PS96, PS97, PS98, PS99, PS100, PS101, PS102, PS103, PS104, PS105, PS118, and PS121) were together in Group II and the remaining three (PS92, PS116, and PS117) were together in Group III. It is important to note that the duplicated genotypes of the cultivar “Cerise” (PS116 and PS117) were placed in the same cluster (Group III).
- *P. hallii* (PS114), one of the diploid progenitors, was completely separated from all the 85 *P. miliaceum* genotypes (allotetraploid).

**FIGURE 5:**
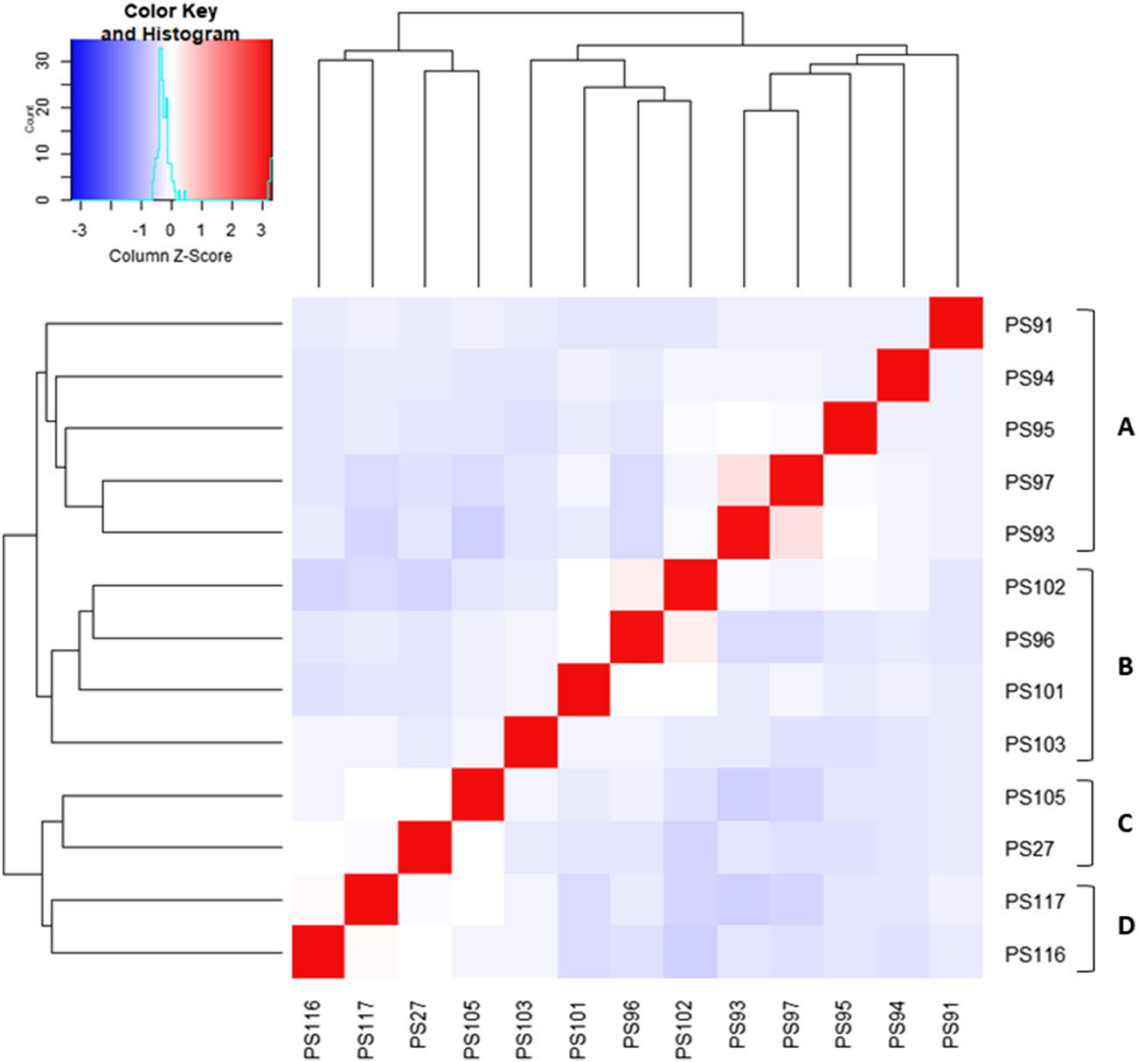
Heatmap and dendrogram showing the genetic relatedness among 13 selected proso millet genotypes estimated using the SNP data. The relatedness among the genotypes was calculated in a kinship matrix which is graphically presented in the figure. The red color and its gradients indicate close association between genotypes, while blue correlates to distantly related genotypes.

In the heatmap and dendogram (Figure 5), we observed that the relative positions of 13 selected genotypes were in 100% match based on their pedigree, and the following are the supporting observations.

- The five genotypes in cluster A were PS91 (Abarr), PS94 (Minco), PS95 (Minsum), PS97 (Cope), and PS93 (Panhandle). Interestingly, all of them are single plant selection of the same landrace “Common white” (Hinze et al., 1978; Hinze & Mann, 1975; NSL65900, 1968; Robinson, 1976; Robinson, 1980).
- Four genotypes in cluster B were PS102 (Rise), PS96 (Dawn), PS101 (Huntsman), and PS103 (Sunrise). Each of Rise and Huntsman has 50% Dawn, and Sunrise has 37.5% Dawn based on their pedigree (Baltensperger et al., 1995; Baltensperger et al., 1997 and Nelson, 1984). Note that Rise (PS102) and Huntsman (PS101) were placed on either side of Dawn (PS96) and Sunrise (PS103) was the farthest from PS96 relative to PS101 and PS102.
- PS27 (PI436626) was the male parent of PS105, cultivar “Plateau” (Santra et al., 2014), and they were next to each other in the heatmap (Cluster C in Figure 5).
- PS116 and PS117, the duplicated samples of the same genotype “Cerise” were plotted next to each other in the heatmap (Cluster D in Figure 5).

These above observations clearly prove that the SNPs reported in our study are non‐random. We understand that, perhaps, a better way of verification is to repeat sequencing of a sub‐set of genotypes and compare the SNPs. Unfortunately, we do not have resources (personnel and fund) to support this additional experiment because the project has ended, and no budget is left. Therefore, we decided not to conduct the additional sequencing.

## 4 DISCUSSION

The sequencing and assembly of the proso millet genome have enabled a new era of proso millet breeding and genetics by enabling the use of high throughput sequencing‐based strategies for discovering genetic markers, which can enhance molecular breeding, quantitative genetic, and population genetic analyses. Here, we identified and scored 972,863 SNPs from 85 accessions of proso millet originated from 23 countries spanning different regions of the globe.

In the current report, the first 10 PCs were found to explain ~33% of the total variation. This finding is consistent with the observations reported by Miao et al. (2019) in several other crop species. They observed that the first 10 PCs in foxtail millet, sorghum, maize, and rice could explain approximately 30%, 40%, 40%, and 15% of the variation, respectively. Therefore, this current result in proso millet is not different from other cereal crops. The PCA analysis of the SNP data grouped the accessions into three distinct clusters. A phylogenetic tree constructed with the SNPs divided the accessions into four discrete groups: Groups I–IV. All 31 but five of the Indian accessions (South Asia) represented Group I. This characteristic of Indian genotypes is consistent with a recent SNP‐based analysis of proso millet population structure using FastStructure which showed that Indian proso millet accessions formed a distinct subpopulation (Johnson et al., 2019). However, our results could not be compared with Li et al. (2021) because not a single genotype between Li et al. and our study is common.

A similar clustering of the North American genotypes based on SSR marker analysis was also reported in our previous study (Rajput & Santra, 2016). In our present study, almost all the North American (16 of 19) accessions were included in group II along with four European accessions. The narrow genetic base of the current US proso millet varieties (16 of 19) is evident from this observation. This has been reported in earlier studies either based on pedigree or molecular marker analysis (Baltensperger, 2002; Rajput & Santra, 2016; Santra et al., 2019). In other words, the majority (20 of 23) of the genotypes in this group have an origin of either Europe (4) or North America (16). In addition, the countries of origin of the four European lines are Hungary (PS17), Romania (PS72), Germany (PS76), and Russian (PS86). This is not unexpected considering the history and origin of proso millet cultivation in the United States. Proso millet was brought to the United States by the German‐Russian immigrants who started cultivating this crop along the eastern Atlantic coast (Habiyaremye et al., 2017; Santra, 2013; Wietgrefe, 1990). Accessions in both Groups III and IV are not represented by a single major country or region. Group III had genotypes from East Asia (China and Taiwan), South Asia, Europe, and North America. Group IV is even most diverse, and the genotypes were from all the regions. The accessions of none of the geographical regions were limited to a single group except the six genotypes from West Asia, which remained together in the single Group IV. This general observation of the distribution of all the accessions from separate geographical regions into multiple groups instead of one is reported in almost all previous studies of genetic relationships based on morphological and molecular markers of different types such as RAPD, AFLP, SSR, and SNPs (Hunt et al., 2011; Khound & Santra, 2020; Santra et al., 2019). This is very much expected considering the history of Asian origin and Eurasian distribution of proso millet, which were elegantly illustrated earlier (Hunt et al., 2011; Lu et al., 2009).

The 972,863 SNPs we identified from a diverse population could serve as a valuable resource for conducting GWAS in proso millet. These SNPs can also be used for marker‐assisted selection (MAS) after proper validation. The relationships among the accessions depicted by the phylogenetic tree could be used for selecting parents for QTL mapping and cultivar development. The findings from this study may encourage researchers working on other minor crops to adopt this approach to identify genetic variants cost‐effectively. Further studies can be conducted on the efficiency of this approach in identifying high‐quality SNPs and other genetic variants in comparison to high‐coverage sequencing.

## CONFLICT OF INTEREST

James Schnable has a significant equity interest in Dryland Genetics, a company seeking to develop new proso millet cultivars. The authors declare no other conflict of interest.

## Supporting information


**Figure S1:** Total number of reads (in million) per individual accession retained after preprocessing.
**Figure S2**: Sequence alignment rates of the 85 proso millet accessions.

**Table S1:** Number of sequenced read bases (Mb), sequence yield and overall alignment rates of 85 proso millet accessions

**Data S1** Supporting Information

## Data Availability

A VCF file containing the SNPs discovered in the current study was deposited at Figshare (https://figshare.com/articles/online_resource/Pm_AllChr_MAF1_Imputed_vcf_gz/20372013).
