# Supplementary material for: SNP discovery in proso millet ( Panicum miliaceum L.) using low‐pass genome sequencing
Source: Plant Direct. 2022 Sep 13;6(9):e447. doi: 10.1002/pld3.447 (PMC9470529; doi:10.1002/pld3.447)
Supplement: Supplementary file 1 — Figure S1: Total number of reads (in million) per individual accession retained after preprocessing. Figure S2: Sequence alignment rates of the 85 proso millet accessions. [file PLD3-6-e447-s002.docx]

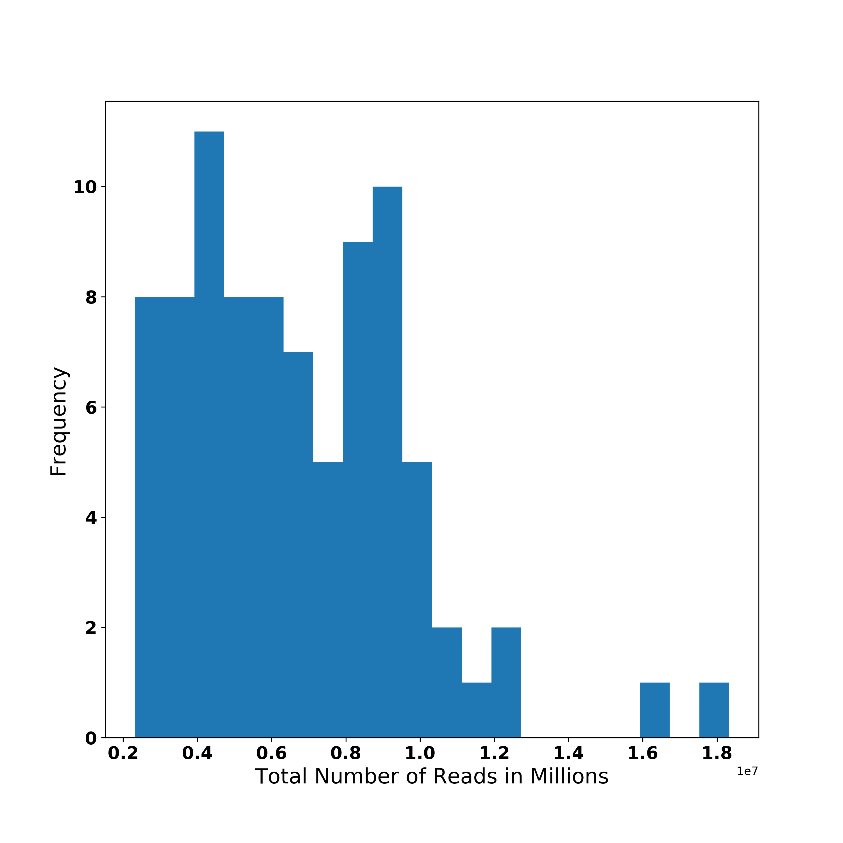

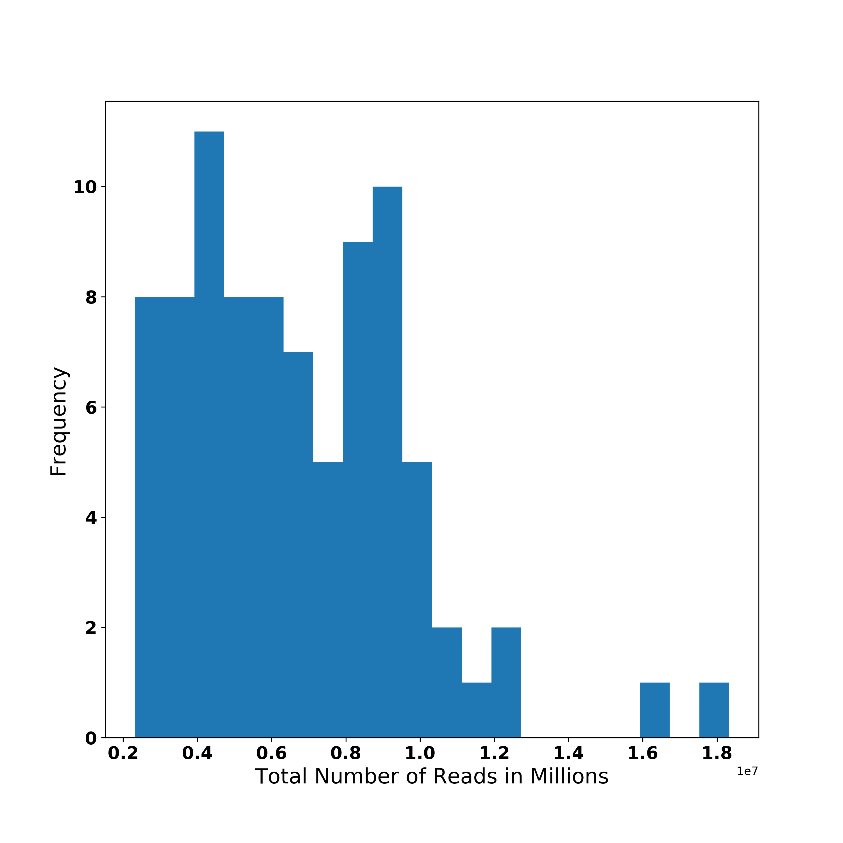


**Supplementary Figure 1**: Total number of reads (in million) per individual accession retained after preprocessing.


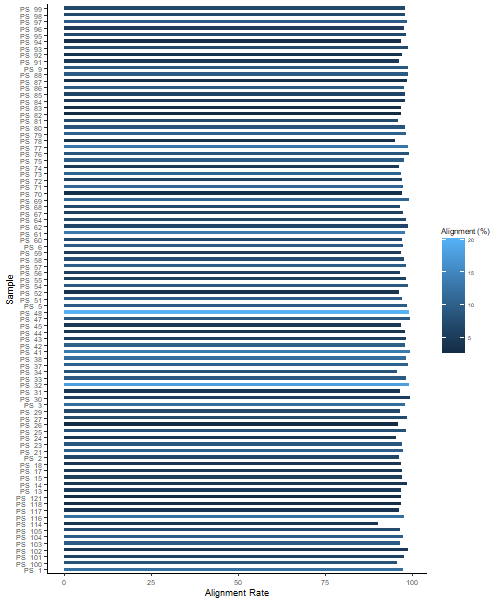


**Supplementary Figure 2**: Sequence alignment rates of the 85 proso millet accessions.
