# Supplementary material for: SNP discovery in proso millet ( Panicum miliaceum L.) using low‐pass genome sequencing
Source: Plant Direct. 2022 Sep 13;6(9):e447. doi: 10.1002/pld3.447 (PMC9470529; doi:10.1002/pld3.447)
Supplement: Supplementary file 2 — Table S1: Number of sequenced read bases (Mb), sequence yield and overall alignment rates of 85 proso millet accessions [file PLD3-6-e447-s003.docx]

**Supplementary. Table 1:** Number of sequenced read bases (Mb), sequence yield and overall alignment rates of 85 proso millet accessions

| **Sample ID** | **Accession no.** | **Origin** | **Geographical region** | **Read bases (Mb)** | **Sequence yield** | **Overall alignment rate** |
| --- | --- | --- | --- | --- | --- | --- |
| PS1 | Ames 11680 | India | South Asia | 1630 | 11,918,906 | 97.42 |
| PS2 | Ames 12008 | India | South Asia | 910 | 6,691,058 | 96.44 |
| PS3 | PI 170595 | Turkey | West Asia | 1580 | 11,549,194 | 98.03 |
| PS5 | PI 177015 | Turkey | West Asia | 1280 | 9,389,918 | 98.62 |
| PS6 | PI 179380 | Turkey | West Asia | 1290 | 9,437,676 | 97.5 |
| PS9 | PI 202295 | Argentina | Others | 1230 | 9,025,298 | 98.86 |
| PS13 | PI 250979 | Serbia and Montenegro | Europe | 670 | 4,891,348 | 96.8 |
| PS14 | PI 251389 | Iran | West Asia | 580 | 4,253,664 | 98.77 |
| PS15 | PI 253421 | Spain | Europe | 770 | 5,629,404 | 97.05 |
| PS17 | PI 289327 | Hungary | Europe | 630 | 4,587,002 | 97.08 |
| PS18 | PI 291364 | China | East Asia | 570 | 4,196,950 | 96.91 |
| PS21 | PI 346942 | Ukraine | Europe | 1360 | 9,896,456 | 97.43 |
| PS23 | PI 427247 | Nepal | South Asia | 1210 | 8,855,166 | 97.26 |
| PS24 | PI 433381 | Taiwan | East Asia | 580 | 4,246,108 | 95.48 |
| PS25 | PI 436623 | China | East Asia | 1100 | 8,028,020 | 98.27 |
| PS26 | PI 436625 | China | East Asia | 370 | 2,721,744 | 96.01 |
| PS27 | PI 436626 | China | East Asia | 980 | 7,171,552 | 98.49 |
| PS29 | PI 463109 | India | South Asia | 970 | 7,092,350 | 96.59 |
| PS30 | PI 463119 | India | South Asia | 730 | 5,352,878 | 99.46 |
| PS31 | PI 463130 | India | South Asia | 570 | 4,157,354 | 96.53 |
| PS32 | PI 463141 | India | South Asia | 2400 | 17,835,146 | 99.3 |
| PS33 | PI 463152 | India | South Asia | 1270 | 9,284,842 | 98.27 |
| PS34 | PI 463163 | India | South Asia | 1160 | 8,461,588 | 95.7 |
| PS37 | PI 463196 | India | South Asia | 1390 | 10,151,306 | 98.91 |
| PS38 | PI 463207 | India | South Asia | 1590 | 11,636,948 | 98.27 |
| PS41 | PI 463240 | India | South Asia | 1860 | 13,575,386 | 99.48 |
| PS42 | PI 463243 | India | South Asia | 1360 | 9,914,998 | 98.09 |
| PS43 | PI 463244 | India | South Asia | 770 | 5,671,938 | 98.24 |
| PS44 | PI 463248 | India | South Asia | 600 | 4,353,666 | 98.05 |
| PS45 | PI 463251 | India | South Asia | 630 | 4,624,916 | 96.95 |
| PS47 | PI 463259 | India | South Asia | 1250 | 9,155,216 | 99.37 |
| PS48 | PI 463263 | India | South Asia | 2750 | 20,105,842 | 99.08 |
| PS51 | PI 463297 | India | South Asia | 1320 | 9,648,598 | 97.33 |
| PS52 | PI 463308 | India | South Asia | 520 | 3,815,256 | 96.28 |
| PS54 | PI 463330 | India | South Asia | 1240 | 9,041,548 | 98.86 |
| PS55 | PI 463341 | India | South Asia | 780 | 5,715,708 | 98.26 |
| PS56 | PI 463352 | India | South Asia | 800 | 5,837,222 | 96.59 |
| PS57 | PI 463363 | India | South Asia | 1300 | 9,549,596 | 98.21 |
| PS58 | PI 463374 | India | South Asia | 1000 | 7,329,484 | 97.64 |
| PS59 | PI 463385 | India | South Asia | 700 | 5,092,564 | 96.85 |
| PS60 | PI 463396 | India | South Asia | 940 | 6,893,232 | 97.26 |
| PS61 | PI 463407 | India | South Asia | 1800 | 13,168,876 | 98.13 |
| PS62 | PI 463418 | India | South Asia | 880 | 6,393,454 | 98.82 |
| PS64 | PI 463440 | India | South Asia | 920 | 6,728,274 | 98.4 |
| PS67 | PI 463473 | India | South Asia | 790 | 5,769,864 | 97.6 |
| PS68 | PI 463484 | India | South Asia | 960 | 7,007,394 | 96.76 |
| PS69 | PI 463495 | India | South Asia | 1490 | 10,861,864 | 99.13 |
| PS70 | PI 463506 | India | South Asia | 450 | 3,258,526 | 97.14 |
| PS71 | PI 476399 | Russia | Europe | 1490 | 10,840,442 | 97.59 |
| PS72 | PI 516181 | Romania | Europe | 800 | 5,896,374 | 97.06 |
| PS73 | PI 517018 | Morocco | Others | 1430 | 10,469,008 | 96.79 |
| PS74 | PI 531400 | Hungary | Europe | 680 | 4,956,320 | 96.3 |
| PS75 | PI 531406 | Czechoslovakia | Europe | 1320 | 9,669,034 | 97.75 |
| PS76 | PI 531407 | Germany | Europe | 1190 | 8,699,316 | 99.21 |
| PS77 | PI 531415 | Russia | Europe | 1720 | 12,620,870 | 98.8 |
| PS78 | PI 531419 | Kenya | Others | 480 | 3,493,842 | 95.05 |
| PS79 | PI 531423 | Poland | Europe | 1420 | 10,333,548 | 98.25 |
| PS80 | PI 531425 | Hungary | Europe | 1160 | 8,449,520 | 98.17 |
| PS81 | PI 531428 | Hungary | Europe | 980 | 7,132,592 | 96.04 |
| PS82 | PI 654404 | Turkey | West Asia | 350 | 2,567,510 | 96.8 |
| PS83 | PI 654404 | Turkey | West Asia | 460 | 3,344,636 | 96.98 |
| PS84 | PI 649377 | South Korea | East Asia | 650 | 4,773,372 | 98.11 |
| PS85 | PI 649380 | South Korea | East Asia | 860 | 6,244,906 | 98.18 |
| PS86 | PI 346935 | Russia | Europe | 1270 | 9,233,664 | 97.78 |
| PS87 | PI 367684 | Australia | Others | 440 | 3,179,824 | 98.66 |
| PS88 | PI 649372 | France | Europe | 1260 | 9,178,100 | 98.84 |
| PS91 | CSR 150 (Abarr) | USA | North America | 630 | 4,663,244 | 96.36 |
| PS92 | PI 649382 | USA | North America | 520 | 3,857,420 | 97.33 |
| PS93 | PI 649383 (Panhandle) | USA | North America | 990 | 7,243,700 | 98.84 |
| PS94 | PI 649384 (Minco) | USA | North America | 450 | 3,309,066 | 96.89 |
| PS95 | PI 649385 (Minsum) | USA | North America | 920 | 6,707,798 | 98.34 |
| PS96 | CSR 152 (Dawn) | USA | North America | 690 | 5,052,704 | 97.77 |
| PS97 | CSR 154 (Cope) | USA | North America | 1300 | 9,554,072 | 98.52 |
| PS98 | NSL 84398 | USA | North America | 950 | 6,947,988 | 98.11 |
| PS99 | PI 536011 | USA | North America | 920 | 6,775,354 | 98.06 |
| PS100 | PI 578073 | USA | North America | 1130 | 8,236,468 | 95.83 |
| PS101 | PI 578074 (Huntsman) | USA | North America | 830 | 6,034,244 | 97.87 |
| PS102 | PI 649386 (Rise) | USA | North America | 620 | 4,549,552 | 98.9 |
| PS103 | PI 583347 (Sunrise) | USA | North America | 1320 | 9,647,156 | 96.56 |
| PS104 | PI 633425 | USA | North America | 1300 | 9,556,212 | 97.52 |
| PS105 | PI 672536 (Plateau) | USA | North America | 960 | 7,025,098 | 96.5 |
| PS116 | PI 170603 (Cerise) | USA | North America | 1650 | 12,058,536 | 97.7 |
| PS117 | PI 170603 (Cerise) | USA | North America | 540 | 3,954,910 | 96.3 |
| PS118 | PI 583347 | USA | North America | 410 | 2,969,082 | 96.88 |
| PS121 | PI 296376 | Canada | North America | 410 | 2,959,966 | 97.03 |
|  |  |  |  | **86,430** | **632,160,746** | **97.70** |
|  |  |  |  | **Total** | **Total** | **Average** |
