# Supplementary material for: SNP discovery in proso millet ( Panicum miliaceum L.) using low‐pass genome sequencing
Source: Plant Direct. 2022 Sep 13;6(9):e447. doi: 10.1002/pld3.447 (PMC9470529; doi:10.1002/pld3.447)
Supplement: Supplementary file 3 — Data S1 Supporting Information [file PLD3-6-e447-s001.pdf]

Response to Editor's Comments

Q.1. The verification of a small subset of the SNPs could make the data more informative.

Response:
We confirmed this based on additional observations of neighbor joining phylogenetic tree and heatmap. From these observations, we concluded that the SNPs identified in this report

For this, a new section (line 265 to 307) and a heatmap (Figure 5) were added.

These additional justifications clearly prove that the SNPs reported in our study are not random but non-random i.e. identified SNPs are authentic and reliable and therefore are reproducible. We understand that another way of verification is to repeat sequencing of a sub-set of genotypes compare the SNPs. Unfortunately, we do not have resources (personnel and fund) to support this additional experiment because the project is ended, and no budget is left. Therefore, we decided not to repeat the additional sequencing.

Q.2. Besides the knowledge of which accessions are closely related, is the SNP data readily available by other researchers? Is the data deposited in JBrowse or something similar? If not, I am not sure the gain for a general user of this data. Thus, information as to how proso millet geneticists can use this information is lacking.

Response: We uploaded a VCF file containing the identified SNPs to figshare.com. The data will be accessible to all proso millet researchers. The link to the file is provided in the revised manuscript under the heading 'Data availability'. The URL of this data file (https://figshare.com/articles/online_resource/Pm_AllChr_MAF1_Imputed_vcf_gz/20372013) is included in the revised manuscript. (line 377-380).

Additional Comments:

- *Incomplete sentence line 48: "....1990) and started early cultivation along the Atlantic coast the cultivation of the crop in North America, which later spread westward into the interior of the continent (Wietgrefe, 1990)."*

  Response: The sentence has been rephrased as, "German-Russian immigrants brought proso millet seeds with them when they migrated to the United States (Habiyaremye et al., 2017; Santra, 2013; Wietgrefe, 1990) and started early cultivation along the Atlantic coast of North America, which later spread westward into the interior of the continent (Wietgrefe, 1990)" (line 44-47).

- *Incorrect statement in line 258: This is because (1) none of 9 the Chinese weedy types of their report were not included in our study.*

  Response: We corrected this as below. We replaced the original sentences by revised sentence (line 327-329) in this revised manuscript.

Original: **"**However, our results could not be compared with Li et al. (2021) where they used 106 proso millet accessions in SNPs-based genetic relationship. This is because (1) none of 9 the Chinese weedy types of their report were not included in our study, and (2) the 97 cultivated accessions of their study, were mainly from China and none from North America and a very few from Europe and South Asia. Whereas, our study included all the North American varieties, a large number of accessions from South Asia, and a fair number of accessions from Europe."
Revised: **"**However, our results could not be compared with Li et al. (2021) because we did find any single genotype common between Li et al. and our study." (Line 327-329)

- *Fig 2, enlarge font size of the figure. The legend in a is also too small.*

  Response: We created a new figure. 2 with enlarged font size. The size of the legend for figure 2(a) was enhanced as well.
